# Prevalence of Metabolic Syndrome and Noncommunicable Disease Risk Factors in Andaman and Nicobar Islands, India, and Their Association With Ayurvedic Psychosomatic Constitution (Prakriti) and Socioeconomic Status: Protocol for a Cross-Sectional Study

**DOI:** 10.2196/81329

**Published:** 2025-12-04

**Authors:** Akashlal M, Azeem Ahmad, Abhayadev A, Lisha S Raj, Manisha M, Arunabh Tripathi, Saroj Kumar Debnath, Bhogavalli Chandra Sekhara Rao, Narayanam Srikanth, Rabinarayan Acharya

**Affiliations:** 1 Regional Ayurveda Research Institute Central Council for Research in Ayurvedic Sciences Sri Vijaya Puram, Andaman and Nicobar Islands India; 2 Central Council for Research in Ayurvedic Sciences New Delhi India; 3 Regional Ayurveda Research Institute Central Council for Research in Ayurvedic Sciences Thiruvananthapuram India

**Keywords:** ayurveda, IDF criteria, international diabetes federation, metabolic syndrome, noncommunicable disease risk factors, Prakriti, socioeconomic status, WHO STEPS instrument

## Abstract

**Background:**

Metabolic syndrome (MetS) comprises several interrelated conditions, including central obesity, insulin resistance, hypertension, and dyslipidemia—all substantially raise the risk of cardiovascular disease and type 2 diabetes mellitus. National Family Health Survey–5 survey findings indicate that the Andaman and Nicobar Islands has a high risk of MetS due to the high prevalence of obesity, hypertension, and diabetes. Ayurveda highlights that an individual’s psychosomatic constitution (Prakriti) plays a key role in determining disease susceptibility, prognosis, and treatment outcomes. Few studies have demonstrated the association and specific Prakriti types with diabetes, hypertension, dyslipidemia, insulin resistance, Parkinson disease, and rheumatoid arthritis. However, the association between MetS and Prakriti remains uninvestigated. Moreover, data on the prevalence of MetS in these islands are limited, and the variations reported in prevalence studies on MetS from mainland India point to challenges in generalizing those findings. Additionally, the unique geography, ethnicity, lifestyle, and health care infrastructure of the Andaman and Nicobar Islands further stress the need for this study.

**Objective:**

This study examines the prevalence of MetS and noncommunicable disease (NCD) risk factors in the South Andaman district and their association with Prakriti and socioeconomic status (SES).

**Methods:**

A cross-sectional study will be conducted with 1000 randomly sampled adult participants from the South Andaman district, Andaman and Nicobar Islands, India. In this study, participants aged 18 years and older who are willing to provide written consent will be included. The exclusion criteria include pregnant women, nonambulant individuals, and those who are unable to undergo Prakriti assessment. MetS will be assessed using the International Diabetes Federation criteria, and NCD risk factors will be recorded via the WHO STEPS (World Health Organization STEPwise approach to noncommunicable disease risk factor surveillance) instrument. Prakriti will be assessed using the Central Council for Research in Ayurvedic Sciences Prakriti Assessment Scale, and SES will be determined using the Modified Kuppuswamy Scale. Data analyses will include descriptive statistics to estimate MetS and NCD risk factor prevalence. Bivariate analyses (chi-square) will explore associations between the variables, quantifying strength with crude and adjusted odds ratios. A multivariable logistic regression will be used to adjust for confounders. Propensity score matching will serve as a sensitivity analysis. Significance is set at an α of .05, using STATA (version 16.1; Stata Corp LLC) software.

**Results:**

The study started in August 2024; as of June 2025, the survey covered 619 participants.

**Conclusions:**

This study will provide crucial data on the prevalence of MetS and NCD risk factors in the South Andaman population and investigate associations between constitutional Prakriti types and SES with MetS and NCD risk factors. Despite the constraints inherent to its cross-sectional design, the research offers essential baseline information to support future studies on both Prakriti and MetS in this unique population.

**International Registered Report Identifier (IRRID):**

DERR1-10.2196/81329

## Introduction

### Background

Metabolic syndrome (MetS) is a cluster of conditions that interlink to each other, such as central obesity, insulin resistance, high blood pressure (BP), and dyslipidemia, which substantially increase the risk of cardiovascular disease and type 2 diabetes mellitus [1]. MetS is also a precursor to many clinical disorders and hence plays an important role in the health situations of the present time [2]. The global prevalence of MetS ranges from 12.5% to 31.4% [3]. Population-based studies in India indicated MetS prevalence of 10% to 30% [4]. Systematic reviews on MetS prevalence in India reveal significant variation influenced by demographic factors, gender, ethnicity, urban-rural differences, diagnostic criteria, study settings, and geographic location [4,5]. This heterogeneity among mainland studies emphasizes the limitation in generalizing their findings to all population groups in India. Further, the Andaman and Nicobar Islands vary from mainland India due to their isolated geography, unique ethnic composition, distinct lifestyle patterns, and differing health care infrastructure [6,7]. Therefore, a population-based study on MetS in the Andaman and Nicobar Islands is needed to address these contextual differences and generate relevant public health data.

The Andaman and Nicobar Islands in India comprise 836 islands in the southeastern part of the Bay of Bengal in the Indian Ocean [8]. The islands’ population was estimated to be 380,581 (males 202,871 and females 177,710) according to the 2011 census [9]. Most (2,38,142 individuals) of this population reside in the South Andaman district [10]. The proportion of people with overweight and obesity in the Andaman and Nicobar Islands is notably higher compared with many other states and the national average in India [11]. Both male and female overweight/obesity increased in the island between 2015-16 and 2019-20 [11]. Moreover, the prevalence of hypertension (25.3% in females and 30.2% in males) and diabetes (17.5% in females and 17.9% in males) is high in this area, according to National Family Health Survey–05 data [11,12]. Since the components of MetS in the Andaman and Nicobar Islands are high, there is a high possibility of the presence of MetS in this population.

According to the Prakriti concept (Ayurvedic psychosomatic constitution) in Ayurveda, every person has a specific body constitution defined by certain psychosomatic features [13]. As per the distinct physical, physiological, and psychological features, people can be divided into 7 different Prakriti types based on Dosha (Vata, Pitta, Kapha, Vata-Pitta, Vata-Kapha, Kapha-Pitta, and Vata-Pitta-Kapha) [13]. Ayurveda suggests that Prakriti can aid in various clinical scenarios like predicting disease susceptibility, determining illness prognosis, and guiding treatment selection [14]. As per the Prakriti concept, diseases manifest differently according to the individual’s constitution [14]. A Pitta-dominant individual is more susceptible to diseases like peptic ulcers, hypertension, and skin disorders [14]. However, a Vata-dominant individual is more prone to musculoskeletal issues like back pain and joint degeneration, and a Kapha-dominant individual faces a higher risk of developing metabolic conditions such as obesity, diabetes, and atherosclerosis [14]. Although Ayurvedic theory proposes that Kapha-dominant Prakriti individuals are inherently more prone to metabolic disorders such as diabetes mellitus, obesity, and insulin resistance, empirical studies are still required to elucidate the potential relationship between Prakriti types and MetS. Based on the concept of Prakriti, several studies have been undertaken to explore the links between specific Prakriti types and various disease conditions. According to Mahalle et al [15], diabetes, hypertension, dyslipidemia, insulin resistance, and inflammatory markers are strongly associated with Vata Kapha and Kapha Prakriti types. Another study by Manyam et al [16] demonstrated a correlation between Vata Prakriti and an increased risk of developing Parkinson disease. Chinthala et al [17] established that those with Vata Prakriti are at a higher risk of rheumatoid arthritis. These findings from research reflect the significance of Prakriti in disease predisposition.

This study examines the prevalence of MetS and noncommunicable disease (NCD) risk factors among the South Andaman population. In addition, the study will also explore the relationship between Prakriti, MetS, NCD risk factors, and socioeconomic status (SES). However, no studies have examined the association between Prakriti and MetS, and no data on the prevalence of MetS in the Andaman and Nicobar Islands is available in the public domain. Hence, the study findings could help develop strategies for preventing and managing MetS and NCDs in the study area. Moreover, the research outcome will guide future research in similar contexts.

### Study Objectives

#### Primary Objectives

The study aims to test whether the prevalence of MetS in the adult South Andaman population is significantly high, and to determine whether different Prakriti types are significantly associated with the occurrence of MetS as well as individual NCD risk factors in this population.

#### Secondary Objectives

The study intends to assess whether the prevalence of major NCD risk factors is significantly high in the adult South Andaman population and to examine whether SES is significantly associated with MetS.

## Methods

### Study Design

This research uses a cross-sectional approach with descriptive and analytical elements. MetS prevalence and risk factors of NCDs among the study population will be measured as part of the descriptive component. On the other hand, the analytical component examines the associations between Prakriti and SES with MetS and NCD risk factors.

### Study Setting

This survey study focuses on the South Andaman district within the Andaman and Nicobar Islands. South Andaman district consists of 30 panchayats and 24 wards, serving as local self-government bodies [18]. The study will select 1000 samples from all these local administrative units (Panchayats and Wards) using a simple random sampling method. The ongoing study was approved and sanctioned by the Central Council for Research in Ayurvedic Sciences (CCRAS) in May 2024, and the total duration of the research is 18 months.

### Participants

The study will include adult individuals aged 18 years or older of either sex who are willing to participate and provide written consent. Pregnant women, nonambulant individuals, and persons who are unable to undergo Prakriti assessments will be excluded from the study. Before enrolling in the study, participants will receive clear and comprehensible verbal and written information regarding the study’s purpose, procedures, potential risks, and benefits. The investigator will ensure that participants fully understand these details. Participation remains entirely voluntary, and participants can withdraw from the study without facing any adverse consequences. A signed and witnessed Informed Consent form is obtained from participants before initiating any study-related activities.

### Variables

The study identifies MetS, NCD risk factors, Prakriti, and SES as outcome variables. MetS is diagnosed based on a combination of waist circumference/BMI, fasting blood glucose, BP, triglycerides (TG), and high-density lipoproteins (HDL) cholesterol levels, following the International Diabetes Federation [19,20]. Central obesity is defined using ethnicity-specific waist circumference thresholds (≥90 cm for males and ≥80 cm for females among Chinese, Malay, and Asian-Indian populations) or a BMI >30 kg/m^2^ [20]. Individuals meeting the criteria for central obesity undergo additional laboratory investigations. A diagnosis of MetS is confirmed if central obesity is accompanied by at least 2 of the following conditions: elevated BP (≥130/85 mm Hg or treatment of previously identified hypertension), elevated fasting blood glucose (≥100 mg/dL, or previously diagnosed type 2 diabetes mellitus), elevated TG levels (≥150 mg/dL or specific treatment for this lipid abnormality), or reduced HDL cholesterol levels (<40 mg/dL in males, <50 mg/dL in females, or specific treatment for this lipid abnormality) [20].

Prakriti is classified into 7 distinct types based on Dosha predominance [13]. Three single Dosha-predominant Prakriti types exist: Vata, Pitta, and Kapha [13]. Additionally, there are 3 dual Dosha-predominant types: Vata-Pitta, Vata-Kapha, and Pitta-Kapha [13]. Finally, 3 Dosha-predominant Prakriti types encompass all 3 Doshas: Vata-Pitta-Kapha [13]. Each individual’s Prakriti is assigned to one of these 7 categories using the standardized Prakriti Assessment Scale developed by the CCRAS [21].

The WHO STEPS (World Health Organization STEPwise approach to noncommunicable disease risk factor surveillance) instrument [22] includes behavioral and physical measurements. Behavioral measurements comprise tobacco and alcohol use, dietary patterns, physical activity, history of raised BP, diabetes, elevated cholesterol, cardiovascular disease, lifestyle advice, and cervical cancer screening (for women). Physical measurements include BP, height, weight, waist circumference, hip circumference, and heart rate. Due to feasibility constraints, biochemical measurements specified in the WHO STEPS questionnaire that are not diagnostic of MetS (such as urinary sodium and creatinine) were excluded.

SES is determined based on the household head’s occupation, education level, and the total family income. Participants are categorized into 5 SES groups: upper class, upper middle class, lower middle class, upper lower class, and lower class. SES is evaluated using the Modified Kuppuswamy Scale [23].

### Data Collection

This study enrolment commenced on August 28, 2024, following approval from the Institutional Ethics Committee of the Regional Ayurveda Research Institute, CCRAS, Port Blair, and the district administrative authorities of South Andaman. A research team comprising a primary investigator, a coinvestigator (Co-I), a senior research fellow, and an assistant is actively engaged in data collection. The team received comprehensive training on data collection methodologies and tools to ensure accuracy and objectivity. Participants are surveyed through direct interviews, laboratory investigations, and physical examinations.

The study team is collaborating with Panchayath/Municipal (local governing body) representatives, Tribal council members, local health authorities, and Anganwadi (mother and childcare center) staff for implementing the study. Data collection methods and questionnaires are thoroughly explained to these representatives before the survey to ensure informed consent from all the participants. This ground-level coordination in the community fosters rapport and effective communication with participants, reducing reluctance, misinterpretation, and social stigma related to data collection and Prakriti classification. All the survey participants are being provided with contact information of the local health care workers, Anganwadi staff, Panchayath/Municipal representatives, Tribal council captains, and the study Investigators to ensure prompt notification and appropriate medical care in case of any complications from venipuncture.

Venous blood samples are drawn from the participant’s forearms. Biochemical parameters, including TG, HDL, and fasting blood sugar, are measured in participants who fast for 12 hours before sample collection. The samples are collected following standard procedures and analyzed using a fully automatic biochemistry analyzer (Snibe Bioassays 240 plus, manufactured in 2021). Standard laboratory procedures are used for all necessary investigations.

BP is measured using an automated BP monitor (Omron digital BP apparatus HEM-8712, manufactured in 2021). Measurements are taken while participants are resting, with their arms placed on a nearby table so the cuff is at heart level. BP is measured 3 times, and the average of the 3 readings is analyzed.

Anthropometric measurements, including height, weight, waist circumference, and hip circumference, are taken using standardized methods. Height is measured with a tension-free, standardized measuring tape, and weight is recorded with a digital weighing machine (Smartcare electronic scale, manufactured in 2021). BMI is calculated by dividing weight (kg) by height squared (m^2^). Waist circumference is measured at the end of a normal exhalation in a horizontal plane, halfway between the lower margin of the ribs and the upper edge of the iliac crest. In contrast, hip circumference is measured at the largest circumference around the buttocks.

### Bias

This study is susceptible to 2 types of biases: selection bias and information bias. Since the survey team conducts door-to-door surveys during working hours, there is a higher likelihood of sampling bias, as the respondents are more likely to be nonworking or older individuals at home during this time. Interviewer bias may arise because cross-verification or secondary review is not feasible while collecting data related to Prakriti and NCD risk factors. Due to subjective variation in the recalling ability of participants, recall bias is also possible in the study.

### Sample Size Calculation

Based on the findings of the Krishnamoorthy et al [4] study, which reported a prevalence of MetS of 30% in Indian adults, a sample size calculation was performed. A relative margin of error of 10% (*d*=0.03) and a 95% confidence level (α=.05) are adopted.

α=.05; hence Z_1–α/2_ = 1.96

p>=(1–q) =30%

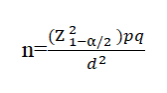



n=897

The study considered a 10% nonresponse; hence, the final sample size will be 897 + 90 = 987 (approximately 1000). The study will take 1000 adult participants from the South Andaman district, Andaman and Nicobar Islands, India. Participants will be chosen through random sampling using the area’s voter list. The voter list is the electoral roll maintained by the Election Commission of India. Each voter has a unique ID, name, gender, age, and address at the polling station level. For South Andaman, the roll is organized by Assembly Constituency, polling stations, and part numbers. After assigning each voter a number, the entire voter roll will be treated as the population frame. The simple random sampling will be performed to select 1000 participants.

### Statistical Analysis

The data will be collected in a single round and subjected to rigorous checks to ensure accuracy and completeness. Any missing or incomplete responses will be addressed through Bayesian imputation, which allows for more robust handling of missingness by incorporating prior information and uncertainty in the estimation process. Following verification and imputation, fundamental descriptive analyses will be conducted to estimate the prevalence of MetS, identify the distribution of NCD risk factors, and describe the baseline demographic characteristics of the study participants. Categorical variables will be summarized as frequency and percentage (n [%]), while continuous variables will be expressed as mean (SD). To explore associations, bivariate analyses will examine the relationship between Prakriti (Ayurvedic constitutional type), NCD risk factors, and SES with the presence of MetS. The chi-square test will be applied to test statistical significance in categorical comparisons. Both crude odds ratios and adjusted odds ratios will be estimated to quantify the strength of associations. A multivariable logistic regression model will compute adjusted odds ratios while accounting for potential confounders. In addition, propensity score matching will be applied as a sensitivity analysis to validate the robustness of the study findings by balancing observed covariates between comparison groups. All statistical tests will be conducted at a 5% level of significance (α=.05). The complete analysis will be performed using STATA software (version 16.1; Stata Corp LLC).

### Data Safety and Monitoring

Using Microsoft Excel software, data collected from the field using paper case record forms (CRFs) is converted into electronic CRFs. Both paper and electronic CRFs will be maintained by the principal investigator (PI). Regular monitoring of data collection and data entry processes will be conducted by either the PI or Co-I throughout the project. The electronic CRFs will undergo cross-verification with paper CRFs by the PI or Co-I to ensure data accuracy.

To safeguard participant confidentiality, documents revealing personal information will only be strictly restricted to project staff. Electronic files will be password-protected, and access to the dataset will be limited to authorized individuals. The statistical department at the CCRAS headquarters will analyze the data. Data will be securely stored for a minimum of 5 years upon completion of the study. An external Data and Safety Monitoring Board will oversee the study to ensure ethical conduct and participant safety.

### Ethical Considerations

The study received ethical clearance from the Institutional Ethics Committee on July 16, 2025 (F. No. 11-5/2024/RARI/IMR-Metabolic Syndrome/264). All study procedures adhered to the ethical principles outlined in the ICMR Ethical Guidelines (2017) and the WMA Declaration of Helsinki. The participant information sheet detailed the purpose of the study, procedures for participation, potential risks and benefits, and provided contact information for the Principal Investigator and Co-Investigators. Field staff were instructed to inform all eligible participants that participation was entirely voluntary and that refusal to participate would not impact their access to health care services. Personal data are handled with strict confidentiality to safeguard individual privacy, and no identifiable information was included in publications without prior informed consent.

## Results

**Figure 1:**
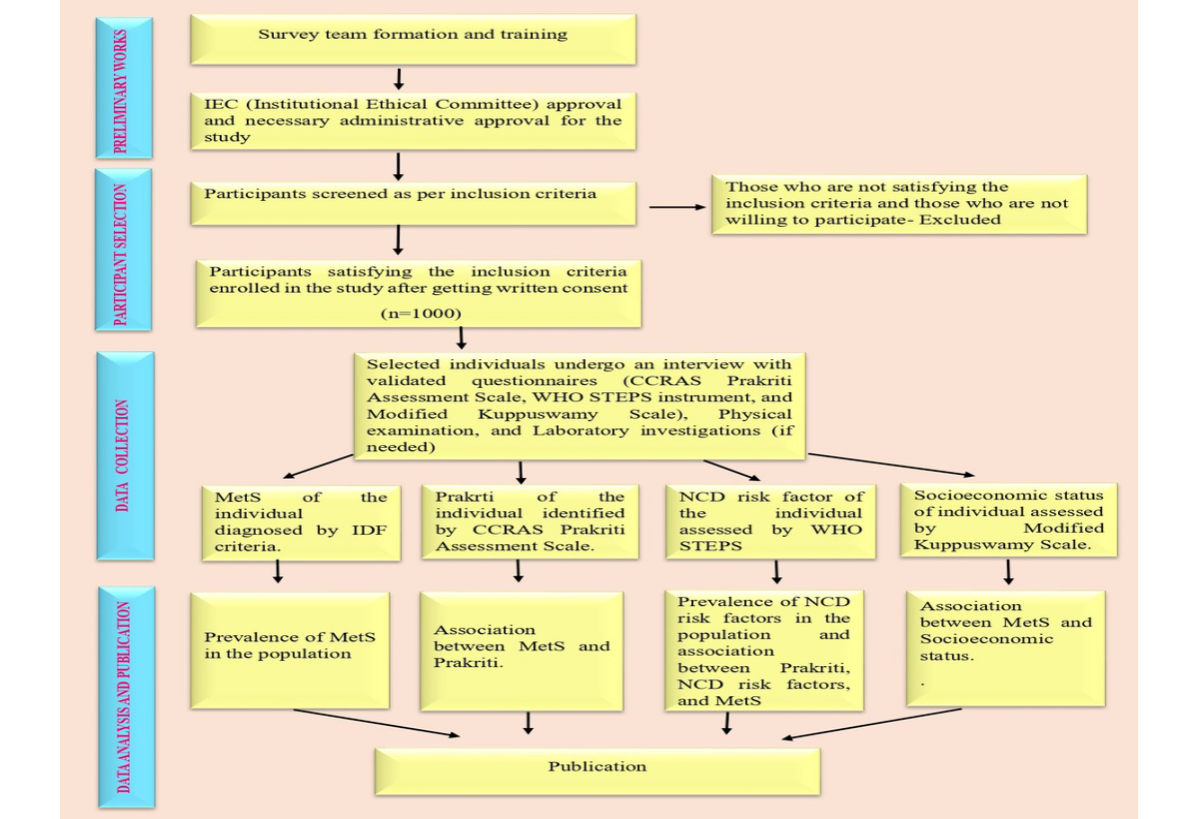
Flowchart showing the methodology of the study. CCRAS: Central Council for Research in Ayurvedic Sciences; IDF: International Diabetes Federation; MetS: metabolic syndrome; NCD: noncommunicable disease; WHO STEPS: World Health Organization STEPwise approach to noncommunicable disease risk factor surveillance.

By June 2025, 619 participants had been enrolled, accounting for 62% of the target sample size, with data entry completed for 480 participants (Figure 1). Of these, 56.46% (n=271) were female, and 43.54% (n=209) were male, with a mean age of 39.88 (SD 14.41) years. Occupational distribution revealed that 40.83% (n=196) were homemakers, while among the employed, 11.25% (n=54) were government employees, 18.54% (n=89) were in the private sector, and 9.79% (n=47) were self-employed. Students comprised 6.25% (n=30) of the sample, and 4.17% (n=20) were unemployed. Educational attainment showed that 42.50% (n=204) had completed primary or secondary education, 24.17% (n=116) had completed high school, and 21.46% (n=103) held a college/university or higher qualification. A small proportion had lower than primary school education (26/480, 5.42%) or no formal schooling (31/480, 6.46%). Marital status analysis indicated that 78.33% (n=376) were married, 16.25% (n=78) were unmarried, and 5.42% (n=26) were widowed.

## Discussion

### Expected Outcomes and Potential Impact

The study is anticipated to provide robust evidence on the prevalence of MetS in the adult South Andaman population, assessing whether it is similar to or higher than in other regions of India. This study’s findings thus help further explore inter-regional variations in the prevalence of MetS in India. Further, drawing on previous research by Mahalle et al [15], which demonstrated strong associations of Vata-Kapha and Kapha Prakriti types with diabetes, hypertension, dyslipidemia, insulin resistance, and inflammatory markers, this study is expected to reveal significant associations between specific Kapha-dominant Prakriti types and the occurrence of MetS, as well as individual NCD risk factors. Evidence indicating that Kapha-dominant individuals exhibit higher BMI, waist-hip ratio, and fasting blood glucose levels supports the hypothesis of a constitutional predisposition to metabolic disorders, which this study aims to substantiate further [24].

A critical limitation identified in the previous study was the lack of comprehensive dietary and lifestyle data documentation [15]. This study addresses this gap to some extent by using the WHO STEPS questionnaire. This scientifically validated tool systematically captures fruit and salt consumption patterns and physical activity levels. This standardized approach will enable exploration of how dietary and lifestyle factors, which are the determinants of NCDs, influence the manifestation of MetS in this unique population.

The study is expected to reveal a substantial burden of major NCD risk factors, including tobacco use, alcohol consumption, physical inactivity, unhealthy diet, and obesity. This expectation is supported by the findings of Ramu et al [6] from a community-based cross-sectional study in rural South Andaman, which demonstrated an alarmingly high burden of behavioral and clinical risk factors of NCD among adults. The outcomes of this study will explore ongoing trends in NCD risk factor burden and assess changes resulting from public health interventions since the previous assessment. SES is anticipated to positively associate with MetS in higher socioeconomic groups in urban study settings, which is consistent with established literature [25].

These outcomes are expected to provide valuable insights into the epidemiological and Prakriti-based determinants of MetS in the South Andaman population. The comprehensive data will provide evidence for future constitutional medicine research and personalized health care approaches. The study will also establish baseline data for longitudinal monitoring of NCD trends and evaluation of preventive program effectiveness in island populations with unique demographic and environmental characteristics.

### Dissemination of Results

A multi-faceted dissemination strategy will be formed to maximize the impact of findings on MetS and Prakriti associations in the South Andaman population. The main manuscript will be submitted to high-impact journals to reach a broad scientific audience. Key results will be presented at national conferences and communicated directly to regional health officials. Information materials like infographics and social media summaries will be created for broader public engagement.

### Strength

The study uses standardized and validated questionnaires to collect data on Prakriti, NCD risk factors, and SES to ensure validity and reliability. This research explores the prevalence of MetS in the South Andaman population, a setting with limited existing data. It also examines potential associations between Prakriti, MetS, NCD risk factors, and SES. The study aims to provide novel insights that contribute meaningfully to the existing literature by investigating these relationships within this relatively understudied population.

### Limitations

In this study, incorporating an objective parameter, such as a biomarker of MetS, could have further strengthened the acceptance of any identified correlations. However, this study did not include such a biomarker due to feasibility constraints. According to Ayurveda, Prakriti is considered a stable constitutional characteristic since birth in an individual; a cross-sectional design can only reveal associations with MetS and cannot establish causal relationships. Likewise, the expected association between socioeconomic status and MetS in this design is limited by the inability to determine directionality or temporal sequence. Therefore, the findings should be interpreted strictly as associations rather than causal evidence.

Additionally, the study is subject to its inherent limitations of cross-sectional designs, including potential selection and information biases. Although statistical methods will be applied to mitigate potential biases, they should be recognized to ensure a transparent and robust interpretation of the results. While the applicability of the findings may primarily pertain to the Indian population, replication in other populations is feasible since Prakriti is not influenced by race or ethnicity [13]. This study did not include the Particularly Vulnerable Tribal Group population in the South Andaman district due to feasibility constraints. These limitations underscore critical knowledge gaps that warrant further investigation in future studies.

## Abbreviations

BP: blood pressure
Co-I: coinvestigator
CCRAS: Central Council for Research in Ayurvedic Sciences
CRF: case record form
HDL: high-density lipoproteins
MetS: metabolic syndrome
NCD: noncommunicable disease
SES: socioeconomic status
PI: principal investigator
TG: triglycerides
WHO STEPS: World Health Organization STEPwise approach to noncommunicable disease risk factor surveillance

## Data Availability

The datasets used or analyzed during this study are available from the corresponding author on reasonable request.
